# Development of Mechanical, Corrosion Resistance, and Antibacterial Properties of Steels

**DOI:** 10.3390/ma14247698

**Published:** 2021-12-13

**Authors:** Marjetka Conradi

**Affiliations:** Institute of Metals and Technology Ljubljana, Lepi pot 11, 1000 Ljubljana, Slovenia; Marjetka.Conradi@imt.si

The total cost and environmental consequences of corrosion problems have become a major challenge to engineers [1]. Steel is known as an important engineering material, mostly it has high corrosion resistance combined with favorable mechanical properties [2,3]. For example, the high corrosion resistance of stainless steel is attributed to the presence of a passive film, which is stable, invisible, thin, durable and extremely adherent and self-repairing [4,5]. However, in many aggressive environments, such as a chloride-ion-rich environment or under physiological conditions, the surface is still observed to suffer from corrosion. For example, reinforced concrete structures require continuous monitoring and maintenance to prevent corrosion of the carbon steel reinforcement [6]. Therefore, in the past two decades, the modification of metallic surfaces by various coatings, organic or polymeric, has become part of an important procedure in enhancing particular surface properties, such as scratch resistance, oxidation, and corrosion [7].

Stainless steels are commonly used materials in biomedical applications also because of their good biocompatibility [8]. However, an increasing number of clinical procedures require the development of materials with superior performance and higher reliability [9]. The major issues in biomedical applications are related to understanding the relationship between the material’s surface properties and the cellular responses, accompanied by the risk of microbial infections [10]. The interaction of nanoscale surface topographies with cells was proven to play a crucial role in the biocompatibility of implants. Various nanoscale surface modifications have been proposed in order to enhance the biocompatibility and antibacterial activity of medical implants [7,11]. Biocompatible polymers, such as hydrophilic polyurethanes, poly(ethylene glycol), and poly(ethylene oxide) brushes, are known to reduce bacterial adhesion by alternating the physicochemical properties of the coating [12,13,14]. Epoxy resins are also extensively used to protect stainless steels because of their good chemical resistance, mechanical properties, strong adhesion with the substrate and corrosion protection by providing an effective physical barrier between the metal and the biological environment [15,16]. Mechanical properties in combination with biocompatibility can be further improved by adding nanoparticles into the epoxy resin, i.e., biocompatible TiO_2_ nanoparticles [7,17]. An alternative approach in modifying surface properties in terms of biocompatibility is treatment with highly reactive plasma, which may alter stainless steel topography, chemistry, and wettability under appropriate treatment conditions [18].

To conclude, the published papers indicate the scientific and technological relevance of the topics covered by the Special Issue. Therefore, this Special Issue represents an important contribution to the broader audience, which will result in an increased number of article readings as well as citations.

## Data Availability

Not applicable.
